# Local alignment vectors reveal cancer cell-induced ECM fiber remodeling dynamics

**DOI:** 10.1038/srep39498

**Published:** 2017-01-03

**Authors:** Byoungkoo Lee, Jessica Konen, Scott Wilkinson, Adam I. Marcus, Yi Jiang

**Affiliations:** 1 Department of Mathematics and Statistics, Georgia State University, Atlanta, GA, USA; 2 Cancer Biology Graduate Program, Emory University, Atlanta, GA, USA; 3 Department of Hematology and Oncology, Emory University, Atlanta, GA, USA; 4 Winship Cancer Institute of Emory University, Atlanta, GA, USA

## Abstract

Invasive cancer cells interact with the surrounding extracellular matrix (ECM), remodeling ECM fiber network structure by condensing, degrading, and aligning these fibers. We developed a novel local alignment vector analysis method to quantitatively measure collagen fiber alignment as a vector field using Circular Statistics. This method was applied to human non-small cell lung carcinoma (NSCLC) cell lines, embedded as spheroids in a collagen gel. Collagen remodeling was monitored using second harmonic generation imaging under normal conditions and when the LKB1-MARK1 pathway was disrupted through RNAi-based approaches. The results showed that inhibiting LKB1 or MARK1 in NSCLC increases the collagen fiber alignment and captures outward alignment vectors from the tumor spheroid, corresponding to high invasiveness of LKB1 mutant cancer cells. With time-lapse imaging of ECM micro-fiber morphology, the local alignment vector can measure the dynamic signature of invasive cancer cell activity and cell-migration-induced ECM and collagen remodeling and realigning dynamics.

Tumor cells are mutated in a multiscale level from a single DNA point mutation to whole chromosome duplication, inversion, and deletion, and thus show large heterogeneity in genotype and phenotype^1^,^2^,^3^. These mutation events transform a normal cell into a cancerous cell, and could eventually result in metastatic disease^4^. Tumor progression, however, is not only impacted by changes in genotype but also by its surrounding tumor microenvironment^5^,^6^,^7^. The extracellular matrix (ECM) is a major component of the tumor microenvironment, where dense and stiff ECM fibers correlate with cancer progression and invasion^6^,^8^,^9^,^10^,^11^,^12^,^13^. Invasive cancer cells dynamically alter the ECM fiber structure using a biochemical enzyme (e.g. matrix metalloproteinases) to degrade^14^, and using the biomechanical force generated from various mechanotransduction signals to condense^6^,^11^,^12^ and realign ECM fibers^8^,^10^,^15^ during tumor progression and metastasis.

Quantitative measures for microscopic ECM fiber structure and alignment are necessary to reveal the dynamic interaction between invading cancer cells and the surrounding ECM environment. Second harmonic generation imaging using a multiphoton microscope allows clear visualization of collagen fibers in 3D^16^. Collagen is a major component of the ECM; methods for automated extraction of collagen fiber segments and quantifying fiber alignment have been developed^17^,^18^,^19^. In addition, several measures, including the orientational order parameter^15^, alignment coefficient^20^, and alignment index^21^,^22^ have been developed to quantify fiber alignment. These measures are based on the fiber angular distribution, where the fiber alignment is 0 if all fibers are randomly distributed, and 1 if all fibers are perfectly aligned. Although these methods quantitatively measure fiber alignment, it is difficult to precisely uncover the cancer cell-induced ECM fiber remodeling, where the fiber structures are spatially heterogeneous. In addition, ECM fiber alignment is often radial to a tumor spheroid surface. We previously developed a local alignment coefficient to address the spatial heterogeneity in the fiber alignment patterns^23^. To capture the spatiotemporal ECM remodeling induced by different cancer cell conditions, we have further developed a novel local alignment vector analysis method to reveal both fiber alignment and alignment direction. This method is based on Circular Statistics^24^. We first demonstrate our method using simulated fibers, and then apply the analysis to post-embedded human non-small cell lung carcinoma (NSCLC) spheroid collagen images.

## Results

### Local alignment vector

**Figure 1:**
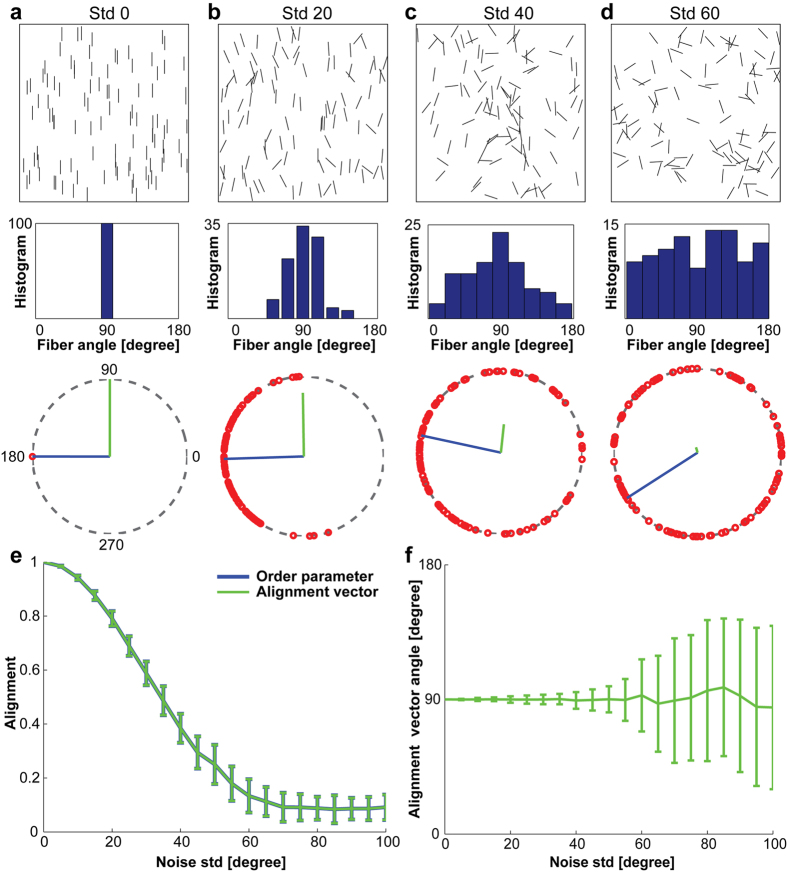
Alignment vector analysis of *in silico* fibers: 100 identical fibers of length 40 are randomly distributed in a simulation box of size 512 × 512. Starting from all fibers that are perfectly aligned to the vertical direction (90 degree), we perturb the fiber orientation by adding an angle that is sampled from normal distribution with zero mean and the standard deviation (std) is (**a**) 0 degree, (**b**) 20 degrees, (**c**) 40 degrees, and (**d**) 60 degrees. The first row shows the configurations of *in silico* fibers, and the second row shows the angular histogram from 0 to 180 degree. The third row shows the fiber angular vectors doubled in angle to map on the unit circle (open red circles), mean resultant vector direction (blue), and the alignment vector (green). (**e**) The length of the alignment vector (green) is exactly the same as that of the orientational order parameter (blue). (**f**) The orientation of alignment vector reflects the direction of fiber alignment. Error bars are standard deviations from 100 replicas of fiber simulations, where fiber orientation perturbation ranges from std = 0 to std = 100 degrees with a 5 degree increment in (**e**) and (**f**).

**Figure 2:**
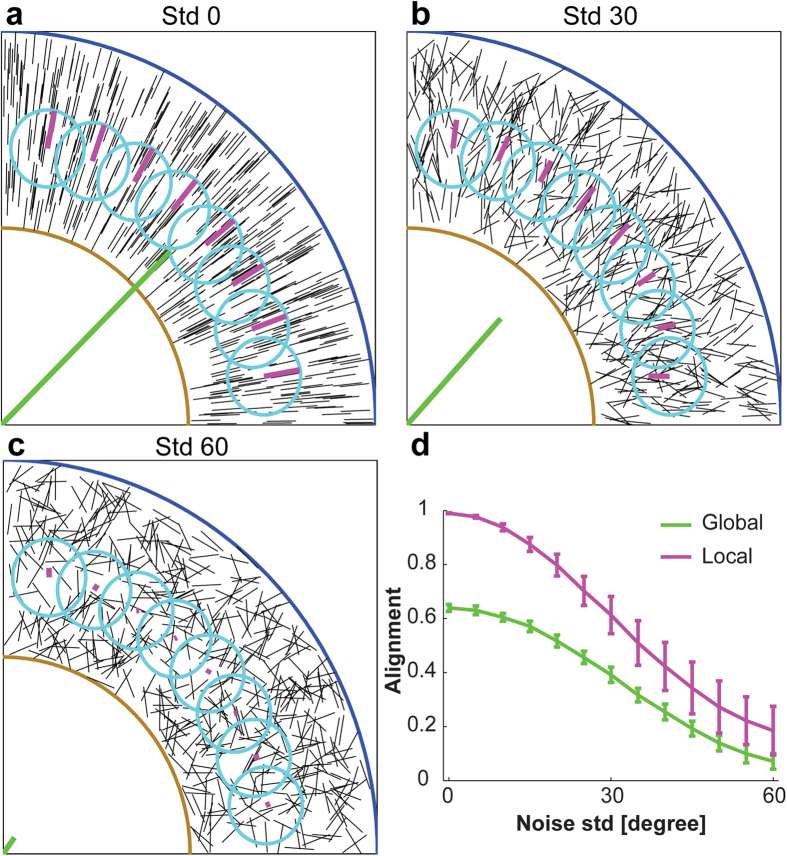
Global vs. local alignment vectors for radially aligned *in silico* fibers normal to a circular boundary (brown). The simulation box is 512 × 512, contains 500 fibers with length equal to 40. Starting from all fibers that are radially aligned to the circular boundary, we perturb the direction by adding an angle, sampled from a normal distribution with zero mean and the standard deviation (std) of (**a**) 0 degree, (**b**) 30 degree, and (**c**) 60 degree. The global alignment vector (green) was calculated for all fibers in the simulation box (or within the blue global circle), and the local alignment vector (magenta) were calculated for the fibers inside of 8 local circles (cyan). (**d**) Comparison between global and local alignment measurement. Error bars are standard deviations from 100 independent replicas of 500 *in silico* fibers with angular perturbations of std = 0 to std = 60 for every 5 degree increment.

Local alignment vector analysis consists of two steps: finding optimal local sampling circle size for a given ECM image, and calculating alignment vector for each local circle. First, we explain how to calculate the alignment vector in Fig. 1, and then describe why we use the local sampling circle in Fig. 2.

As an illustration, we show fibers (black straight lines in Fig. 1) randomly distributed in a simulation box of 512 × 512, where each fiber length is 40 in an arbitrary unit. Starting from a perfectly ordered case where all fibers are aligned to the vertical direction (90 degree), we perturb each fiber orientation by adding a random angle, sampled from a normal distribution with zero mean and a standard deviation (std) value that is 0 degree (Fig. 1a), 20 degree (Fig. 1b), 40 degree (Fig. 1c), and 60 degree (Fig. 1d). Each simulation box has a total of 100 fibers. The histograms of fiber angles, in the second row of Fig. 1a–d, show the fiber alignment from perfectly aligned (Fig. 1a) to almost random (Fig. 1d). Then, we double each fiber angle (*α*: 0–180 degree) to map them onto the circle (*θ*: 0–360 degree), where *θ* = 2*α*. The third row in Fig. 1a–d shows the doubled fiber angles (open red dots) on the unit circle (dotted gray circle). By definition in Circular Statistics^24^, the mean resultant vector of 100 fiber angles is the vector sum of all fiber angular vectors divided by the total fiber number (Eq. 1). The fiber angular vector, (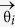), is defined as the unit vector from the center of the unit circle (x = 0, y = 0) to the angular point on the unit circle (x_i_ = cosθ_i_, y_i_ = sinθ_i_), where θ_i_ = 0–360 degrees. The mean resultant vector (MRV) is then


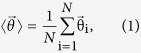


where *N* is the total number of fibers. Finally, we divide the angle of the mean resultant vector by half while keeping its vector length to result in the alignment vector, shown as the green line in the unit circle in the third row in Fig. 1a–d. The alignment vector length reflects the degree of alignment: 1 for the perfectly aligned fibers in Fig. 1a, and decreases as the noise std angle increases in Fig. 1b–c, to almost 0 in Fig. 1d. For the almost randomly distributed fibers in Fig. 1d, the alignment vector length is close to zero, and thus the alignment direction was not clearly revealed. The blue unit vector in the circle at the third row in Fig. 1a–d was added to clearly visualize the angular vector direction of the mean resultant vector.

The alignment vector length on the unit circle, which measures the alignment of fibers, can vary from 0 to 1. If all fibers are perfectly aligned, the alignment vector length is 1, while the alignment vector length is close to 0 if they are randomly distributed. The alignment vector is equal to 0, when the mean resultant vector length is 0, e.g. 45 and 135 degree in the fiber angular histogram (*α*: 0–180 degree), or 90 and 270 degree in the unit circle (*θ*: 0–360 degree) for a two fiber sample case, as an example.

To verify whether our alignment vector length correctly measured the fiber alignment, we compared our alignment vector length with orientational order parameter μ:^15^


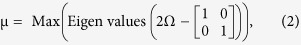


where 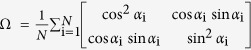 and *N* is the total number of fibers.

We use the same simulation box of 100 fibers as in Fig. 1a–d. We varied the noise std angle from 0 to 100 degree with a 5 degree increment, each with 100 replicas. Figure 1e appears as only one line because the alignment vector length (green) is exactly equal to the orientational order parameter (blue), meaning that the alignment vector is the accurate measurement of fiber alignment as the orientational order parameter did. Additionally, the alignment vector offers the direction of fiber alignment that other alignment measurements^15^,^20^,^21^,^22^ could not. Figure 1f shows the alignment vector angle for the simulated fibers in Fig. 1e. The average angle of alignment vectors accurately reflects the fiber alignment direction, the increasing angular perturbations showing as increasing error bars. The alignment direction is difficult to address without using Circular Statistics^24^. We demonstrated this point in supplementary Fig. 1.

To better address the heterogeneous and irregular fiber morphology, we calculate the alignment vector of fibers in a local sampling circle. Figure 2 illustrates typical ECM fiber patterns around a tumor spheroid^8^,^10^,^12^, where fibers (black) are radially aligned, normal to the circular tumor boundary (brown). Note that tumor boundaries can have complex geometry, and our method applies equally well to any geometry. Without loss of generality, we illustrate the method of selecting a sampling circle using radially aligned fibers with a quarter of circular tumor. The simulation box is 512 × 512, and the length of each fiber is 40. Fibers have different degree of radial alignment normal to the circular tumor boundary, from perfectly aligned with std = 0 degree (Fig. 2a), std = 30 degree (Fig. 2b), to std = 60 degree (Fig. 2c). Figure 2d shows the comparison between two different alignment vectors: the global alignment vector (green) for all fibers (global circle radius = 512), and the local alignment vector (magenta) for the fibers located inside of local sampling circles (local circle radius = 50). Figure 2d shows the comparison between global and local measurements of fiber alignment, for which we examined fiber patterns with varying the noise std from 0 to 60 degrees in 5 degree increment and 100 independent replicas for each case. The local alignment vector more accurately calculates the fiber alignment than the global alignment vector in heterogeneous fiber patterns, because the latter averages out alignment of different orientations. For example, the local alignment reaches 1 for the perfectly aligned fibers (std = 0 degree), while the global alignment is only about 0.6. In addition, the global alignment vector is pointing to 45 degree, while eight different local alignment vectors more accurately present the fiber alignment directions as normal to the boundary (Fig. 2a). Even with increasing angular perturbation (Fig. 2b–c), the local alignment vector consistently provides both alignment value and direction more precisely than the global alignment vector. Therefore, our local alignment vector better reflects the fiber alignment in more complex and heterogeneous fiber geometries.

### Local alignment vector of *in vitro* NSCLC tumor spheroids

Next we explain how to properly choose the local sampling circle size using collagen images from *in vitro* NSCLC tumor spheroid invasion experiments. If the local sampling circle is too small, then too few fibers are included for the calculation, and thus the alignment is not well defined. If the local circle is too large, then the local alignment vector loses the local feature. As an extreme case, it will be the global alignment vector.

We use two different non-small cell lung cancer (NSCLC) cell lines (H1299, H157) with various perturbations to liver kinase B1 (LKB1) and microtubule affinity regulating kinase 1 (MARK1) pathways^23^,^25^,^26^ to demonstrate the discriminatory power of the local alignment vector measure. Liver kinase B1 (LKB1), also known as a serine-threonine kinase (STK11), is a tumor suppressor^26^,^27^,^28^. Mutations and inactivation of LKB1 are observed in different cancer types, such as melanoma, lung, breast, and cervical cancers^28^,^29^,^30^,^31^,^32^. LKB1 is one of most frequently mutated genes in lung adenocarcinoma with missense and truncating LKB1 mutations primarily in its kinase domain^33^,^34^. LKB1 directly phosphorylates and activates AMP-activated protein kinase (AMPK), and its family member MARK1^25^,^26^,^35^. MARK1 goes on to inhibits focal adhesion kinase (FAK) to regulate collagen remodeling and cancer cell motility^23^,^36^,^37^. Therefore, LKB1 is the upstream regulator of AMPK and MARK1, and play a key role in cancer cell invasion and ECM remodeling and realignment.

Ten different test conditions in the two cell lines, summarized in Table 1, were chosen to investigate the mechanism underlying how LKB1 regulates invasive migration. In each test case, cancer cells were grown as tumor spheroids, and then embedded in a 2 mg/ml collagen gel. Second harmonic generation (SHG) collagen images and cell images were taken simultaneously at 0, 6 and 21 hours for H1299, and 0 and 24 hours for H157. First, we illustrate our local alignment vector analysis of one SHG image of collagen. Then we show the analysis results of full z stack images at various time points after seeding each tumor spheroid into a collagen gel.

**Figure 3:**
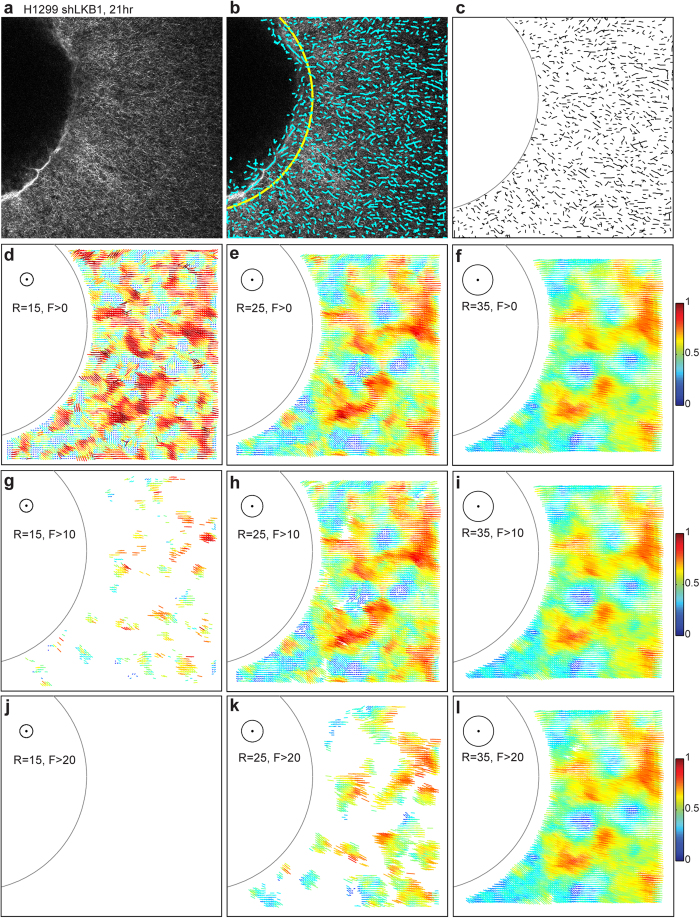
Local alignment vector of H1299 shLKB1, the first z stack image (the bottom of a collagen gel) at 21 hour. The image size is 512 × 512 pixels, and each pixel is 0.83 μm. (**a**) Multiphoton microscopy image using the second harmonic generation signals, (**b**) Extracted fiber segments (cyan color) using CT-FIRE. A circular tumor boundary (yellow) is manually selected. (**c**) Quantized fiber segments. (**d–l**) local alignment vectors for varying two different parameters: local circle size (the circle radius, R = 15, 25, 35 pixel) and minimum fiber segment number (F > 0, 10, 20). Inset figures illustrate each local circle and two different parameters. A colorbar is attached to show color-coded alignment vectors, where the alignment vector length changes from 0 to R in each figure, and the alignment value changes from 0 to 1 after the local circle is converted to the unit circle.

Figure 3a shows a typical SHG collagen image of the H1299-shLKB1 spheroid, which is stably depleted of LKB1 at 21 hours after seeding the spheroid into a 2 mg/ml collagen gel. We extract collagen fiber segments (cyan, Fig. 3b) from the image using curvelet transform fiber extraction software (CT-FIRE)^17^, and manually select a circular tumor boundary (yellow arc in Fig. 3b). In most cases, the spheroids are not perfectly spheroidal, hence the tumor boundaries in 2D images are not perfectly circular, especially when cancer cells actively invade outwards. Without losing much information, we manually fit the circular tumor boundary for this set of images.

The extracted fibers vary in length, including short specs that come from segmentation noise. In addition, the contribution of a fiber to the alignment measure is proportional to the length of the fiber, and thus a long fiber is equivalent to many segments of short and straight fibers. To address these points, we quantize the extracted fiber segments using a filtering length, which should be long enough to filter the fiber extraction noise, but short enough to quantify the length of the fibers. Also, a curvy fiber can be split into many 5-pixel long straight segments. We use a filtering length of 5 pixels (Fig. 3c). We then apply the local sampling circles for each SHG image with 5 pixel intervals in x and y, which result in 10,609 (103 × 103) local alignment vectors per each image. We exclude local alignment vectors, when their local sampling circles cross the image boundary or the tumor boundary, or when their local sampling circles contain fewer quantized fiber segments than a minimum fiber number. Figure 3d–l show local alignment vectors for three different local sampling circle sizes (figure insets, radius R = 15, 25, 35 pixels, respectively) and three different minimum fiber numbers (F > 0, 10, 20). Local alignment vectors in Fig. 3d–l are color-coded according to their normalized alignment vector lengths. The normalized alignment vector length in the image varies from 0 (randomly distributed) to 1 (perfectly aligned). With the decreasing local sampling circle size, the local alignment vectors reflect more precisely the heterogeneous fiber alignment patterns, when we compare between Fig. 3f and 3e, and between 3e and 3d. But at the same time the sensitivity of the local alignment vector decreases because smaller local sampling circles contain fewer quantized fiber segments, when we compare between Fig. 3d-3g, and between Fig. 3g-3j. Supplementary figures S2–S5 illustrate the effect of local sampling circle size on both local alignment and quantized fiber segment count of 7 different local circle sizes for all z stacks of SHG images, and the procedure to select the optimal local circle size (R = 25 pixel).

### Local alignment vector analysis of *in vitro* H1299 tumor spheroids

**Figure 4:**
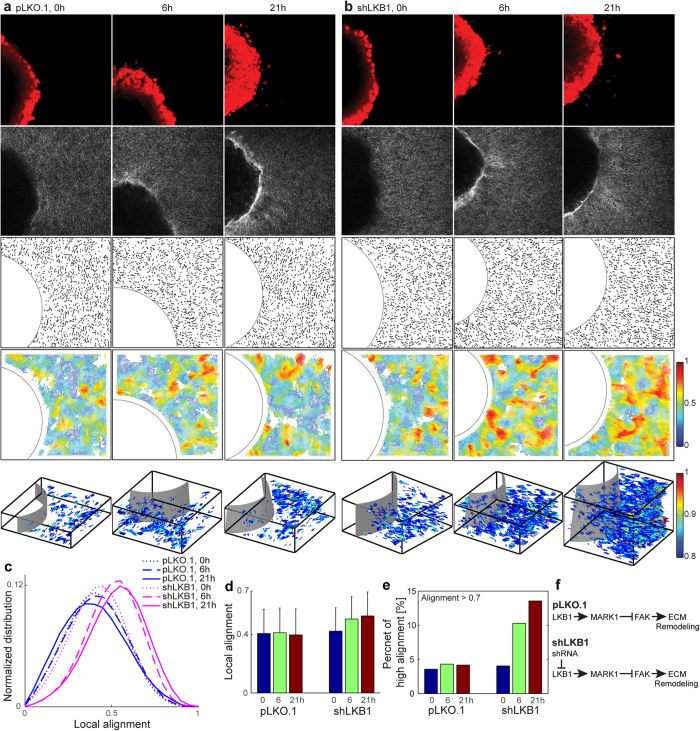
Local alignment vector analysis of *in vitro* H1299 tumor spheroids in a collagen gel. (**a**) pLKO.1 control case, (**b**) shRNA knocked down LKB1 case (shLKB1) at 0, 6, and 21 hour after seeding a tumor spheroid into a 2 mg/ml collagen gel. The first row in (**a** and **b**) is fluorescent microscopy images to visualize tumor spheroids, dyed using CellTracker Red; the second row is multiphoton microscopy images to visualize collagen fibers using second harmonic generation signals; the third row is quantized fiber segments with manually selected circular tumor boundary after extracting collagen fibers using CT-FIRE; the fourth row is local alignment vectors. These 2D fiber images are the first z stack images (the bottom of collagen gel). The fifth row is local alignment vectors of all z stacks in 3D, where we plot only high alignment vectors (alignment > 0.8). For the clear visualization, we magnify 4 times in Z, e.g. X:Y:Z = 1:1:4. (**c**) Normalized local alignment distribution using all z stack data. The local alignment (**d**) and the high alignment ratio over the whole z stacks (**e**) were calculated. (**f**) LKB1 signaling pathway for control case (pLKO.1) and shRNA knocked down case (shLKB1).

H1299 pLKO.1 (control) and shLKB1 (LKB1 depleted) tumor spheroids were embedded a collagen gel (Fig. 4a,b). Multiple z-stack multiphoton microscopy images at 0, 6, and 21 hours post-embedding were collected. The first row in Fig. 4a-4b shows tumor cells, dyed using CellTracker Red, the second row shows the corresponding SHG microscopy images of collagen, the third row shows the quantized collagen fiber segments, and the fourth row shows the local alignment vectors. These 2D fiber images are the first z stack image (the bottom of collagen gel). The fifth row shows the local alignment vectors of all z stacks in 3D, where we plot only the high alignment vectors with alignment values larger than 0.8, for the clear visualization. Alignment vectors in 3D are color-coded according to their alignment values.

Local alignment vectors of pLKO.1 show no significant change over time. The local alignment vectors of shLKB1, in comparison, show a significant increase of high alignment over time. Normalized local alignment distribution curves using all z stacks clearly show that the alignment of shLKB1 increases over time (Fig. 4c), while the distribution curves of pLKO.1 show no significant shift over time. The amount of shift is quantified in Fig. 4d. We also quantify the ratio of strong-alignment, defined as the volume ratio of alignment value greater than a threshold value. A threshold value of 0.7 is used in Fig. 4e. In shLKB1, the ratio increases from 4% at 0 hour to 10% at 6 hour, and then 14% at 21 hour, while in pLKO.1, the ratio shows no change at around 4% at 0, 6, and 21 hour. Supplementary figure 6 shows the ratio of strong-alignment at various alignment threshold values. Supplementary video 1 shows eight different z stack multiphoton images with quantized fiber segments for H1299. Supplementary video 2 shows high-alignment vectors (greater than 0.8) in 3D (with z axis magnified by 4) by rotating the azimuth angle to better visualize the alignment vectors outside the tumor boundary.

### Local alignment vector analysis of *in vitro* H157 tumor spheroids

**Figure 5:**
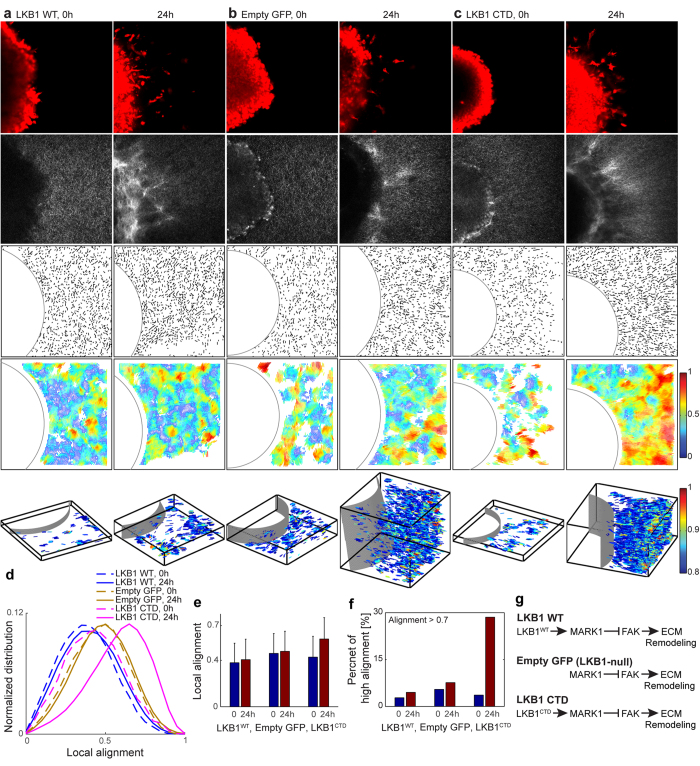
Local alignment vector analysis of *in vitro* H157 tumor spheroids in a collagen gel. Spheroids of (**a**) LKB1^WT^, (**b**) Empty GFP (LKB1-null), (**c**) LKB1^CTD^ (C terminal domain only) are imaged at 0 and 24 hours after seeding into a 2 mg/ml collagen gel. The first row in (**a–c**) are fluorescent microscopy images of tumor spheroids, dyed using CellTracker Red; the second row are SHG images of collage fibers; the third row is quantized fiber segments with manually selected circular tumor boundary after extracting collagen fibers using CT-FIRE; the fourth row is the local alignment vectors. These 2D fiber images are the first z stack images (the bottom of collagen gel). The fifth row are local alignment vectors of all z stacks in 3D, where we plot only the high alignment vectors (alignment > 0.8). For clear visualization, scale in Z is magnified 4 times, i.e., X:Y:Z = 1:1:4. (**d**) Normalized local alignment distribution using all z stack data. The local alignment (**e**) and the high alignment ratio over the whole z stacks (**f**) were calculated. (**g**) LKB1 signaling pathway for LKB1^WT^, Empty GFP, and LKB1^CTD^ case.

**Figure 6:**
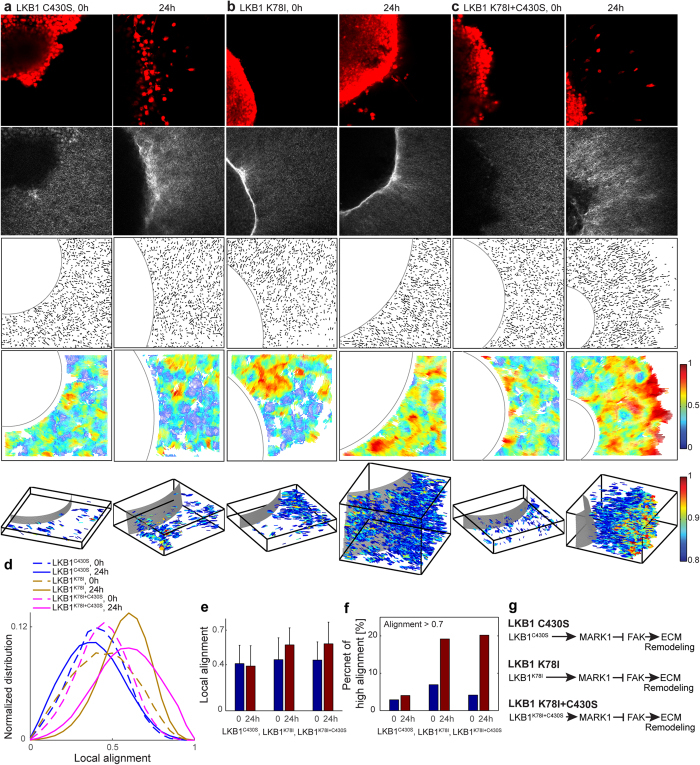
Local alignment vector analysis of *in vitro* H157 tumor spheroids in a collagen gel. (**a**) LKB1^C430S^ (farnesylation mutant), (**b**) LKB1^K78I^ (kinase-dead mutant), (**c**) LKB1^K78I + C430S^ (double mutant in kinase domain and farnesylation domain) cases at 0 and 24 hour after seeding a tumor spheroid into a 2 mg/ml collagen gel. The first row in (**a–c**) is multiphoton microscopy images to visualize tumor spheroids, dyed using CellTracker Red; the second row is multiphoton microscopy images to visualize collage fibers using second harmonic generation signals; the third row is quantized fiber segments with manually selected circular tumor boundary after extracting collagen fibers using CT-FIRE; the fourth row is local alignment vectors. These 2D fiber images are the first z stack images (the bottom of collagen gel). The fifth row is local alignment vectors of all z stacks in 3D, where we plt only high alignment vectors (alignment > 0.8). For the clear visualization, we magnify 4 times in Z, e.g. X:Y:Z = 1:1:4. (**d**) Normalized local alignment distribution using all z stack data. The local alignment (**e**) and the high alignment ratio (**f**) over the whole z stacks were calculated. (**g**) LKB1 signaling pathway for LKB1^C430S^, LKB1^K78I^, and LKB1^K78I + C430S^ case.

**Figure 7:**
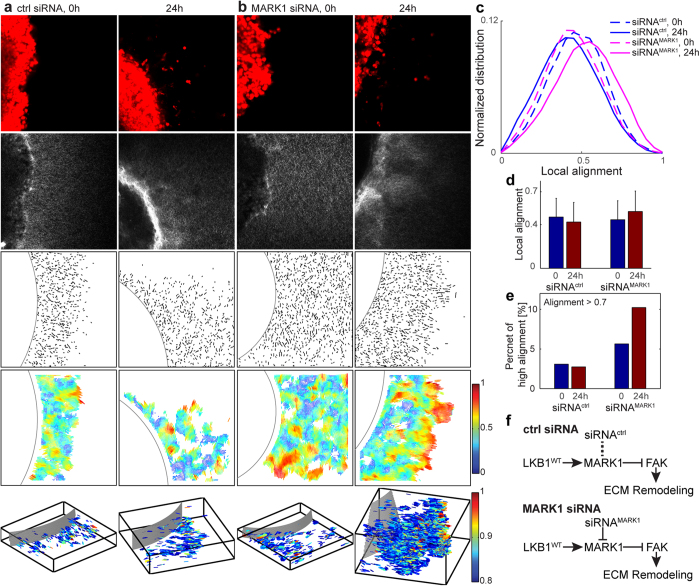
Local alignment vector analysis of *in vitro* H157 tumor spheroids in a collagen gel. (**a**) LKB1^WT^ + siRNA^ctrl^, (**b**) LKB1^WT^ + siRNA^MARK1^ cases at 0 and 24 hour after seeding a tumor spheroid into a 2 mg/ml collagen gel. The first row in (**a** and **b**) is multiphoton microscopy images to visualize tumor spheroids, dyed using CellTracker Red; the second row is multiphoton microscopy images to visualize collagen fibers using second harmonic generation signals; the third row is quantized fiber segments with manually selected circular tumor boundary after extracting collagen fibers using CT-FIRE; the fourth row is local alignment vectors. These 2D fiber images are the first z stack images (the bottom of collagen gel). The fifth row is local alignment vectors of all z stacks in 3D, where we plot only high alignment vectors (alignment > 0.8). For the clear visualization, we magnify 4 times in Z, e.g. X:Y:Z = 1:1:4. (**c**) Normalized local alignment distribution using all z stack data. The local alignment (**d**) and the high alignment ratio over the whole z stacks (**e**) were calculated. (**f**) LKB1 signaling pathway for LKB1^WT^ + siRNA^ctrl^ and LKB1^WT^ + siRNA^MARK1^ case.

To further demonstrate the power of the local alignment vector in discriminating subtle features of ECM remodeling during cancer invasion, we examined different perturbations to the LKB1 and MARK1 pathway^23^,^25^,^26^,^36^. We embedded LKB1-null H157 tumor spheroids in a 2 mg/ml collagen gel for the following conditions: LKB1^WT^ (wild type, Fig. 5a), Empty GFP (LKB1-null, Fig. 5b), LKB1^CTD^ (deletion mutation and contains only C-terminal domain of LKB1, Fig. 5c), LKB1^C430S^ (point mutation at farnesylation domain of LKB1, Fig. 6a), LKB1^K78I^ (a point mutation in the kinase domain of LKB1, Fig. 6b), LKB1^K78I+C430S^ (double mutations at kinase and farnesylation domain of LKB1, Fig. 6c), LKB1^WT^ + siRNA^ctrl^ (siRNA with no target, Fig. 7a), and LKB1^WT^ + siRNA^MARK1^ (siRNA targeting MARK1, Fig. 7b).

We imaged cells with multiphoton microscopy in multiple z stacks at 0 and 24 hours post-embedding. In Figures 5, 6 and 7, the first rows show tumor cells, dyed using CellTracker Red, the second rows show the corresponding SHG microscopy images of collagen, the third rows show the quantized fiber segments, and the fourth rows show the local alignment vectors. These 2D fiber images are the first z stack image, the bottom of tumor spheroid embedded collagen gel images. The fifth rows are the local alignment vectors of all z stacks in 3D, where we plot only the high local alignment vectors with alignment values larger than 0.8, for the clear visualization. The alignment vectors in 3D are color-coded according to their alignment values, offering direct visualization of the spatial heterogeneity of the ECM alignment in 3D.

The local alignment vectors for LKB1-null (Empty GFP) show no significant change over time. Adding wild type LKB1 (LKB1^WT^) made the normalized distribution of local alignment shift to the low alignment region compared to Empty GFP (in Fig. 5d-5e). This suggests that LKB1^WT^ results in less aligned ECM fibers, which is consistent with the LKB1-MARK1 regulatory pathway in Fig. 5g^23^,^35^,^36^,^38^. Interestingly, LKB1 kinase-dead mutants (LKB1^CTD^, LKB1^K78I^, LKB1^K78I+C430S^) show significantly increased local alignment, shown in the 4^th^ and 5^th^ rows of Fig. 5c and Fig. 6b–c. We quantify this increased alignment as a shift to the high alignment region over time (Fig. 5d–e and Fig. 6d–e). In comparison, the LKB1 mutant in non-kinase domain (LKB1^C430S^) does not significantly alter the collagen morphology (the 4^th^ and 5^th^ rows in Fig. 6a), and no significant shift of the local alignment distribution over time (Fig. 6d–e.) The ratio of strong-alignment shows that LKB1 kinase-dead mutants exhibit significant increase in the ratio from 4% at 0 hour to 29% at 24 hours for LKB1^CTD^, from 7% at 0 hour to 19% at 24 hour for LKB1^K78I^, and from 4% at 0 hour to 20% at 24 hour for LKB1^K78I+C430S^. But for LKB1^WT^, Empty GFP, and LKB1^C430S^, the ratio shows no significant change over time. Figures 5g and 6g illustrate the corresponding regulatory pathways of these different perturbation conditions. These results strongly suggest that disruption of LKB1 kinase activity plays an integral role in ECM realignment during lung cancer cell invasion.

In addition, the alignment distribution for siRNA knocked-down MARK1 (H157 LKB1^WT^ + siRNA^MARK1^) shows a significant shift to high alignment in 24 hours compared to the control (H157 LKB1^WT^ + siRNA^ctrl^) in Fig. 7a–d. The strong-alignment ratio for the siRNA knocked-down MARK1 also increases from 5.6% at 0 hour to 10.2% at 24 hour, while the control does not, from 3.1% at 0 hour to 2.7% at 24 hour. These results indicate that MARK1 is closely related to the cancer cell-induced ECM fiber remodeling activity, which is similar to prior observations^23^,^25^,^35^,^36^.

We demonstrate that our local alignment vector can discriminate quantitatively the spatial heterogeneous ECM fiber alignment pattern and examine the ECM remodeling activity between invasive and non-invasive cancer cell conditions. Interestingly, we observe high alignment vectors even at 0 hour, as in H157 LKB1^K78I^ (Fig. 6b). To embed a tumor spheroid into the collagen gel, not locate on the top of the gel, a preparation process is required, for 1 hour. H157 LKB1^K78I^ case shows that some invasive cancer cells mechanically interact with surrounding collagen fibers within 1 hour.

Supplementary video 3, 5, 7 show eight different z stack multiphoton images with quantized fiber segments for H157 LKB1^WT^, Empty GFP, LKB1^CTD^ (video 3), LKB1^C430S^, LKB1^K78I^, LKB1^K78I+C430S^ (video 5), LKB1^WT^ + siRNA^ctrl^, LKB1^WT^ + siRNA^MARK1^ (video 7). Supplementary video 4, 6, 8 show high alignment vectors (greater than 0.8) in 3D by rotating azimuth angle to clearly visualize the alignment vectors with the tumor boundary for H157 LKB1^WT^, Empty GFP, LKB1^CTD^ (video 4), LKB1^C430S^, LKB1^K78I^, LKB1^K78I+C430S^ (video 6), LKB1^WT^ + siRNA^ctrl^, LKB1^WT^ + siRNA^MARK1^ (video 8). The Supplementary figure 7 shows the strong-alignment ratio at various alignment threshold values.

## Discussion

We developed a novel local alignment vector method using Circular Statistics^24^, which can quantify both alignment amplitude and alignment direction for heterogeneous fiber distributions. The method works well for a circular boundary of a tumor spheroid, but also applies to more complex geometries, where previous averaged and directional-independent measures would fail. Two features of the local alignment vector analysis are important: the small filter length to quantize the fibers, and the optimal local circle size to define locality. Together they address the heterogeneous local features of collagen fibers.

We demonstrate our new method through analyzing *in silico* fibers with a circular tumor boundary and *in vitro* collagen fibers resulting from cancer cell invasion using lung cancer cell lines with various perturbations in the LKB1-MARK1 pathway. Both the *in silico* and *in vitro* fiber analysis results show that the local alignment vector reveals the spatiotemporal dynamics of heterogeneous collagen fiber remodeling.

Our quantitative local alignment vector measures discriminate between strong-ECM-interacting group of invasive cancer cells and weak-ECM-interacting group of non-invasive cancer cells. The strong-ECM-interacting group includes knocked-down LKB1 (H1299 shLKB1), LKB1 kinase domain mutants (H157 LKB1^CTD^, LKB1^K78I^, LKB1^K78I+C430S^), and siRNA knocked-down MARK1 (H157 LKB1^WT^ + siRNA^MARK1^) cell lines, which all show significant changes in collagen fiber morphology and alignment over time. The weak-ECM-interacting group includes active LKB1 (H1299 pLKO.1), rescued LKB1 (H157 LKB1^WT^), LKB1 non-kinase domain mutant (LKB1^C430S^), and the control silencing case (H157 LKB1^WT^ + siRNA^ctrl^), which do not have a significant change of the local alignment over time, nor high alignment vectors from the tumor spheroid boundary outward. Because ECM fiber alignment is a consequence of mechanical interaction between cells and collagen fibers, we suggest that the alignment is a manifestation of the mesenchymal polarity of the cancer cells. The discriminating capacity between these groups, and the subtle discrimination within the groups, help to interpret the invasiveness of these cell lines and their ability to promote mesenchymal polarity during invasion. The FAK inhibiting role of LKB1 is consistent with the fact that cells with knocked-down LKB1 could significantly remodel the ECM^36^,^37^. The LKB1 kinase domain mutants show that LKB1 kinase activity is crucial for FAK-mediated cell adhesion and collagen remodeling and realignment^23^,^37^.

When we embed a tumor spheroid in a collagen gel, we have to wait for an hour for the spheroid to settle in the gel before taking the first image at time 0. Otherwise, the tumor spheroid may not be embedded completely in the gel. During this one hour preparation time, the more invasive cell lines could have started interacting with the collagen, resulting in some high alignment vectors even in the initial 0 hour image, shown in the strong-ECM-interacting group. Hence we quantify the collagen fiber remodeling by comparing the difference between the initial alignment and the subsequent alignment measurements at 6 and 21 hours for H1299 cell line, and at 24 hours for H157 cell line.

To address potential concerns that we might not have considered enough spatial regions for collagen alignment quantification, we note that invasive cancer cells migrate approximately 10–100 microns within 24 hours, safely within the region that we imaged and analyzed (425 × 425 μm, or 512 × 512 pixels, 1 pixel is 0.83 μm). As the spheroid and cancer invasion does not have any orientational preference, any region outside the invading spheroid should offer the same information. In addition, the number of alignment vectors in one z stack image is 10,609 (in every 5 pixel interval in x and y). Even after excluding the alignment vectors whose sampling circle cross the image boundary or tumor boundary and those with fewer fiber segments than the minimum fiber constraint, the total number of the alignment vector for all the z stack images in one experiment is in the order of 10 thousand. This large number helps to offer sufficient statistics to clearly distinguish the difference between inactively ECM fiber remodeling cases (e.g. pLKO.1) and actively ECM fiber remodeling cases (e.g. shLKB1).

One limitation of the current method is the manual process to find the tumor boundary in the z-stack images. We can use rather standard boundary segmentation methods, e.g. computational geometry based on generalized convex hull^39^, to automate this step. Other preprocessing steps, including choosing the optimal local circle size and the fiber filtering length, can also be automated. It is interesting to note that the optimal local circle size depends on the image resolution and the efficiency of the fiber segmentation software (CT-FIRE). With higher resolution images, we can reduce the segmentation noise, and thus choose smaller local circle size and shorter fiber filtering length.

The alignment vectors are in 2D. When we stitch together the z-stacks images, the local alignment vectors can also provide fiber alignment distribution in 3D (Figs 4, 5, 6 and 7, and in the supplementary video 2, 4, 6, 8). This ability is based on the small quantization fiber length with properly addressing the heterogeneous fiber local features using the optimal local circle. With the recent advances in imaging technology, e.g., super resolution microscopy that dramatically increases the spatiotemporal resolution^40^, intravital second and third harmonic generation microscopy that can visualize cancer cell-induced ECM remodeling activity *in vivo*^41^, more quantitative full-3D ECM imaging *in vivo* should be readily available. It means our method will have more direct application to 3D collagen quantification in a wide range of contexts.

In addition, 3D traction force microscopy can measure the stress applied from each cell to surrounding ECM^42^,^43^. Combining with individual cancer cell trajectories in ECM, local alignment vector can provide necessary information to distinguish different cancer cell migration modes, directional cell migration which may not realign ECM fibers^44^ *vs* cell-induced mechanically realigning ECM fibers^10^,^12^,^15^,^20^. Integrating with these new methods and data, more comprehensive understanding of the interaction between invasive cancer cells and surround ECM will be possible in the near future. Our local alignment vector is compatible with these new data and provides a complement and quantitative tool toward a more comprehensive understanding of the interaction between metastatic cancer cells and the surrounding ECM.

The ECM fibers in histology slides^12^ showed similar features to our *in vitro* collagen fibers, and thus our local alignment vector should rather easily apply to the histology slides where ECM fibers are imaged. *C*ancer cells *in vivo* show more complicated migrating behavior in 2D and 3D ECM microenvironments^45^,^46^,^47^. In addition, the tip cell or leader cell phenotype is different from the followers in collective cancer cell invasion^48^,^49^. Furthermore, invasive cancer cells demonstrate plasticity in migration modes depending on their local environment. Many cancer cells also secret MMPs to degrade ECM at the higher traction stress^50^. The complexity of the multifaceted interactions means that a single parameter cannot fully describe cancer cell invasion. Rather, a system of description for both cancer cells and their microenvironment would be required. Our local alignment vector provides one additional quantitative tool to uncover the dynamic signature of the interaction of cancer cells and ECM.

## Methods

### NSCLC culture and cell line generation

H1299 and H157 human NSCLC cells (ATCC, Manassas, VA) were cultured in Roswell Park Memorial Institute (RPMI-1640) media supplemented with 10% fetal bovine serum and 100 units/mL of penicillin/streptomycin, and maintained at 37 °C and 5% CO_2_. The H1299 stable LKB1 knockdown (shLKB1) cells were created as previously described^23^,^37^. Briefly, cells were created using lentiviral infection using LKB1 specific shRNA constructs from Open Biosystems (Rockford, IL). pLKO.1 empty vector was used as a control. Transduced cells were selected using puromycin (2 μg/ml). H157 GFP-LKB1 cells were created as previously described^23^. Briefly, LKB1 wildtype and various protein truncates were subcloned into the pBabe-puro vector and stably expressed in H157 (LKB1-null) lung cancer cells. Puromycin (1 μg/ml) was used to select for cells expressing the plasmid.

### Generating 3-D tumor spheroids

Spheroids were formed as previously described^23^. Briefly, H1299 and H157 cells were plated in a Spheron Nunclon 96 well plate (Thermo Scientific, Waltham, MA) at a cell density of 1.5 × 10^4^ cells/ml. After 2–4 days, spheroids were collected and resuspended in 2.0 mg/ml collagen type I (Advanced Biomatrix, San Diego, CA), then plated in a 35 mm glass bottom dish (*In Vitro* Scientific, Sunnyvale, CA) for multiphoton microscopy.

### Multiphoton imaging of spheroids

Spheroids of H1299 shLKB1 and pLKO.1 or H157 stable cells were dyed using 1 μM of Red CellTracker (Invitrogen). The H1299 stable spheroids were imaged at 0, 6, and 21 hours post-invasion, and H157 stable spheroids were imaged at 0 and 24 hours post-invasion, using a standard upright Zeiss Axio Examiner Z1 microscope with 20x water immersion objective (1.0 NA DIC (UV) VIS-IR). The second harmonic generation (SHG) signal was obtained using a bandpass 380–430 nm cube. To image the cells stained with Red CellTracker, a bandpass of 570–610 nm cube with a long pass of 550 nm was used. Images were taken with a Coherent Chameleon Verdi laser at 790 nm wavelength. Each multiphoton microscopy image is 512 × 512 pixels in size (425 × 425 μm); each pixel is 0.83 μm. Z-stack images were taken with a 1 μm interval.

### CT-FIRE

Curvelet Transform–FIbeR Extraction software (CT-FIRE)^17^ was downloaded and installed from the web site of the Laboratory for Optical and Computational Instrumentation (LOCI) at the University of Wisconsin-Madison, loci.wisc.edu/software/ctfire. Win64 Beta 1.2.1 version was used to extract collagen fibers from the second harmonic generation microscopy images. CT-FIRE uses the curvelet transform^51^ for the image denoising process and the fiber extraction algorithm^18^, which describe the detailed procedures and parameters to extract fiber segments from the images.

### Local alignment vector

Fibers are extracted from SHG images using CT-FIRE and quantized using a threshold filtering length. The local alignment vectors are calculated in three steps: first, map the fiber angles (*α*: 0–180 degrees) onto the unit circle (*θ*: 0–360 degree) by doubling each angle; second, calculate the mean resultant vector (MRV)^24^ of all fibers; and third, divide the angle of the mean resultant vector by 2 to map it back to the upper plane (0–180 degree). We show below that the local alignment vector length is equal to the orientational order parameter.

Local alignment vector length = MRV length = 


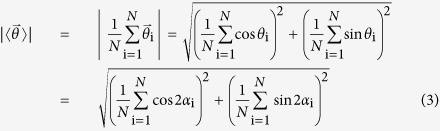


The orientational order parameter (μ) is defined as the maximal eigenvalue of the following matrix *A*^15^.


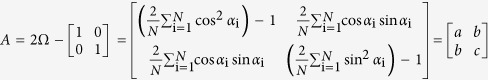


We solve for the maximum eigenvalue of *A*:


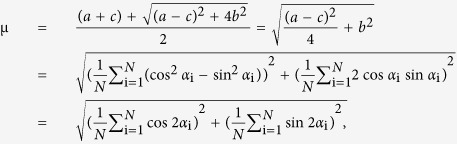
 [Disp-formula eq5]

is exactly the same as 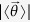 in equation (3).

## Additional Information

**How to cite this article**: Lee, B. *et al*. Local alignment vectors reveal cancer cell-induced ECM fiber remodeling dynamics. *Sci. Rep.* **7**, 39498; doi: 10.1038/srep39498 (2017).

**Publisher's note:** Springer Nature remains neutral with regard to jurisdictional claims in published maps and institutional affiliations.

## Supplementary Material

Supplementary Dataset

Supplementary Movie 1

Supplementary Movie 2

Supplementary Movie 3

Supplementary Movie 4

Supplementary Movie 5

Supplementary Movie 6

Supplementary Movie 7

Supplementary Movie 8

Supplementary Information

## Figures and Tables

**Table 1:** Human non-small cell lung carcinoma (NSCLC) test conditions.

NSCLC Cell lines LKB1 activity	LKB1/MARK1 perturbation	Image collected time (hour)
H1299 Active LKB1	pLKO.1 (control)	0, 6, 21
	shLKB1 (shRNA knocked-down LKB1)	
H157 Inactive LKB1	LKB1^WT^ (wild type)	0, 24
	Empty GFP (LKB1-null)	
	LKB1^CTD^ (C-terminal domain only)	
	LKB1^C430S^ (farnesylation mutant)	
	LKB1^K78I^ (kinase-dead mutant)	
	LKB1^K78I + C430S^ (double mutant)	
	LKB1^WT^ + siRNA^ctrl^ (siRNA control)	
	LKB1^WT^ + siRNA^MARK1^ (siRNA targeting MARK1)	
