# Supplementary figures and images for: Local alignment vectors reveal cancer cell-induced ECM fiber remodeling dynamics
Source: Sci Rep. 2017 Jan 3;7:39498. doi: 10.1038/srep39498 (PMC5206731; doi:10.1038/srep39498)

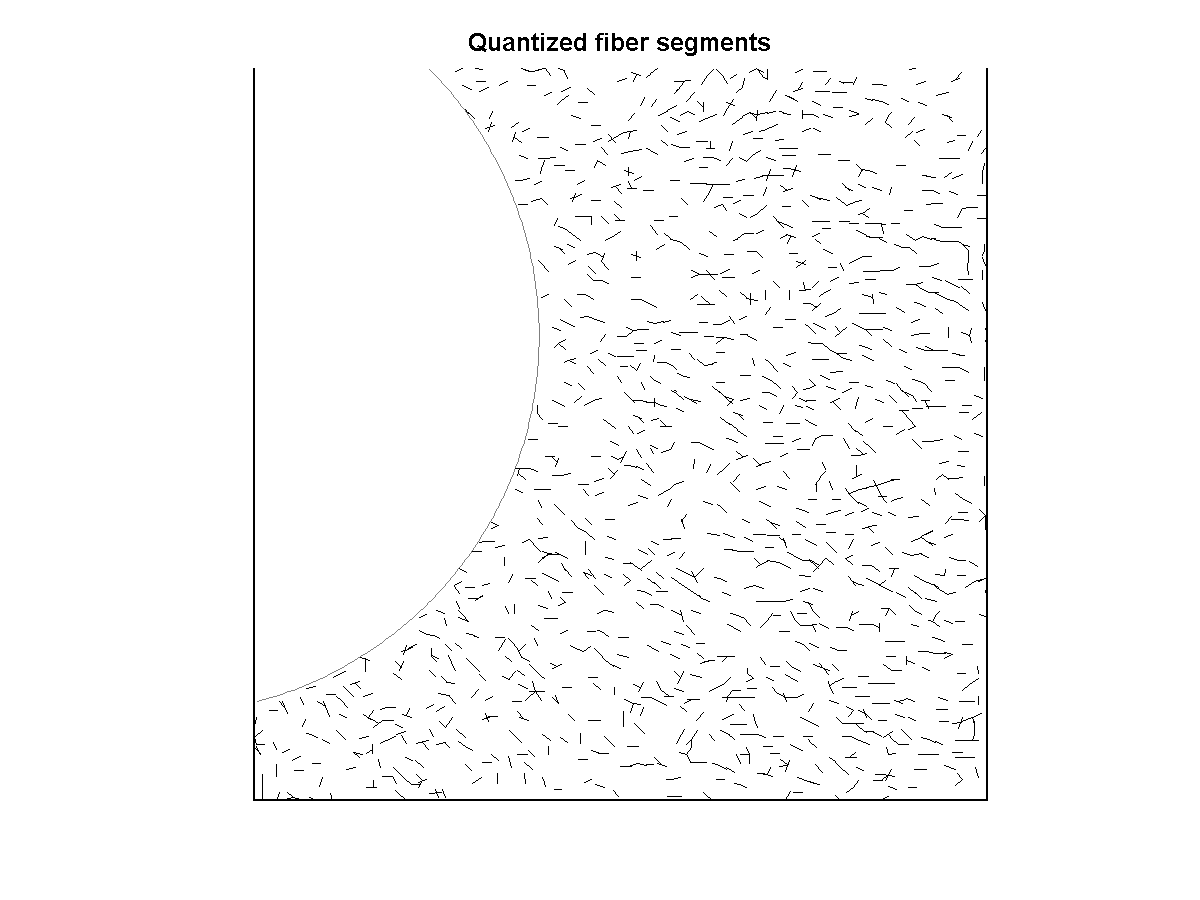

Supplement: Supplementary Dataset [file srep39498-s1.zip › Step1_Fig3c.tif]

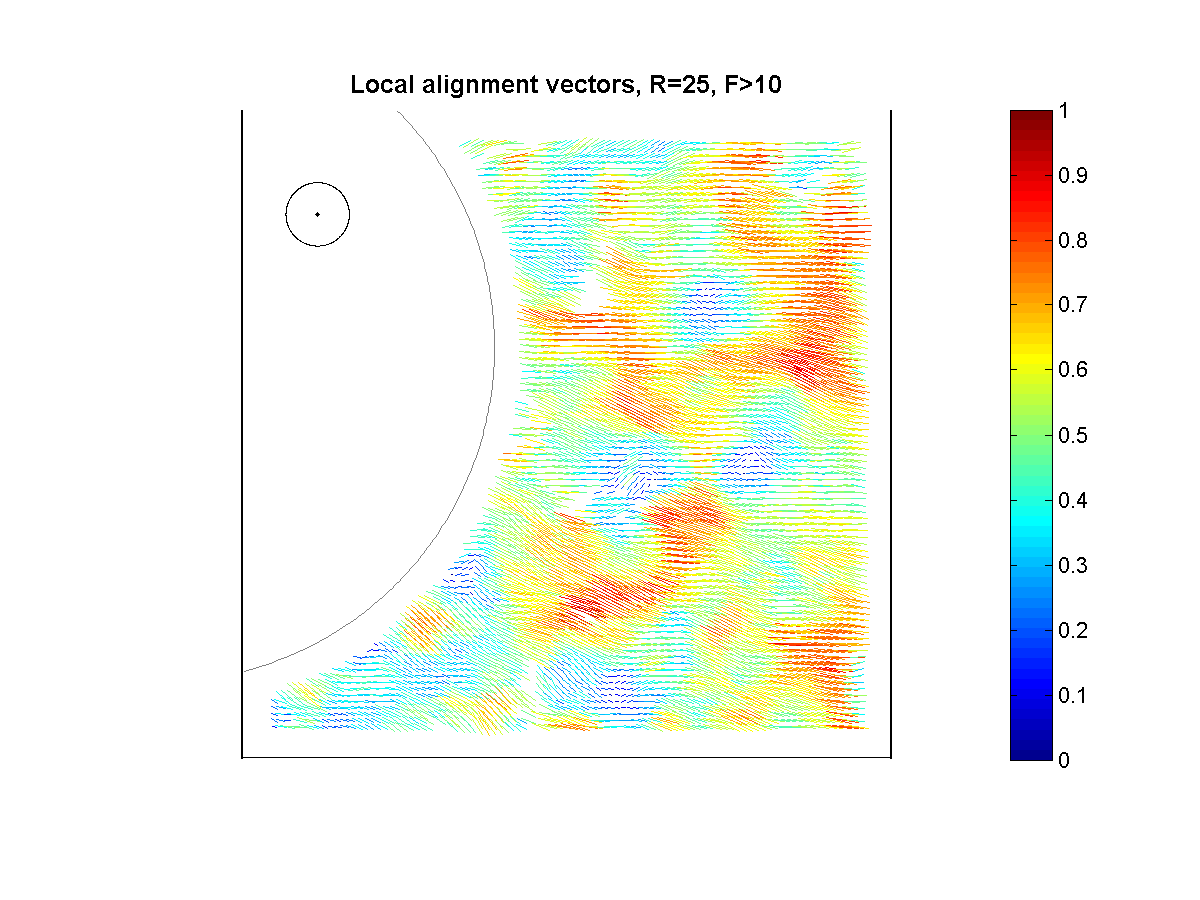

Supplement: Supplementary Dataset [file srep39498-s1.zip › Step4_Fig3h.tif]

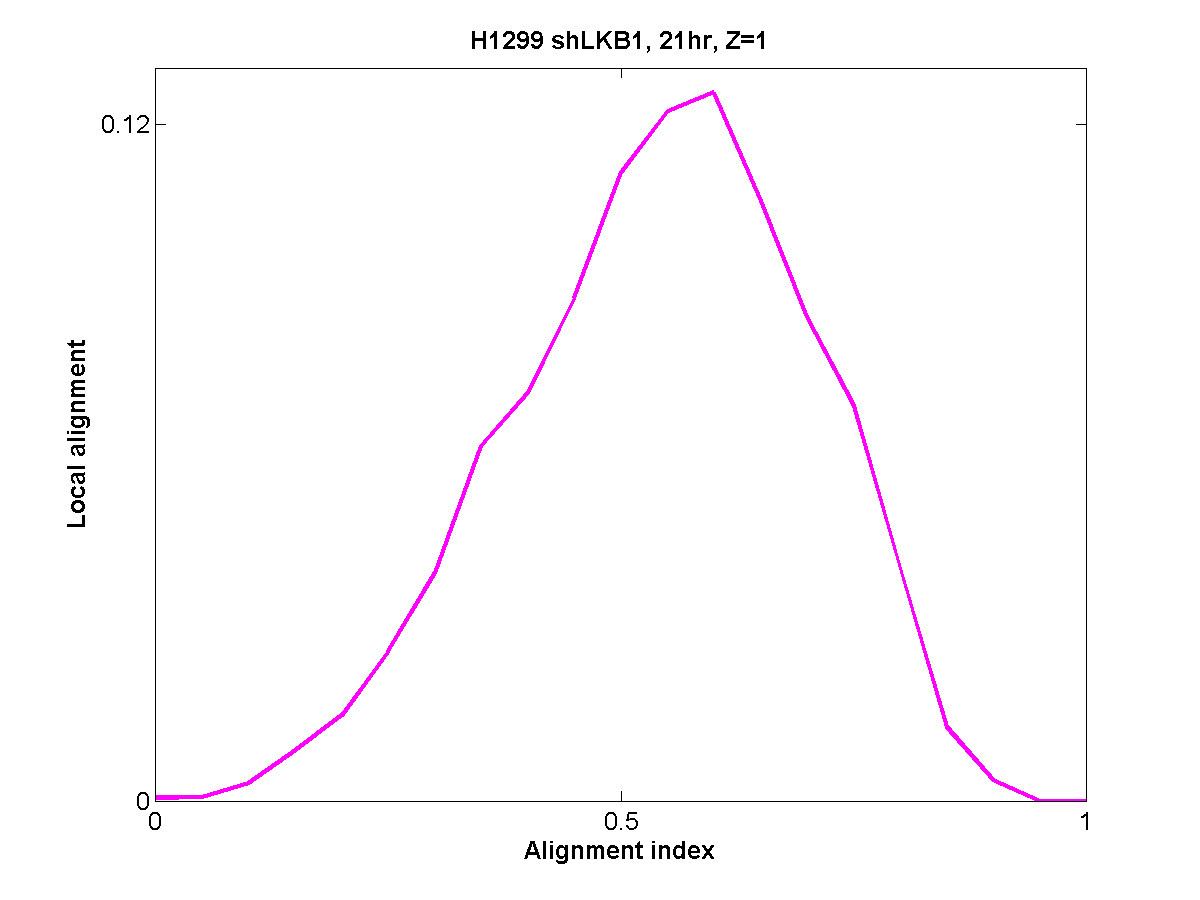

Supplement: Supplementary Dataset [file srep39498-s1.zip › Step5.tif]

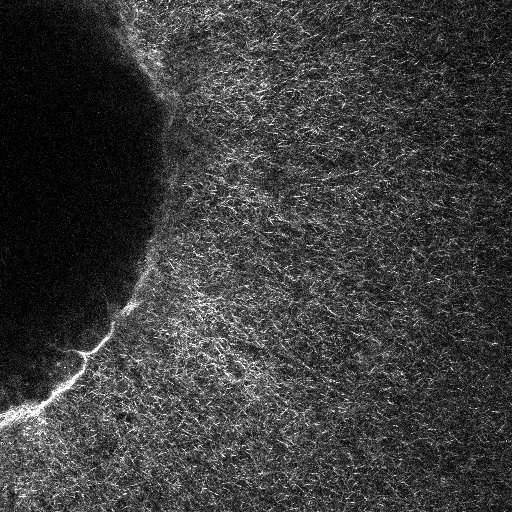

Supplement: Supplementary Dataset [file srep39498-s1.zip › shLKB1_21hr_z01c2.tif]

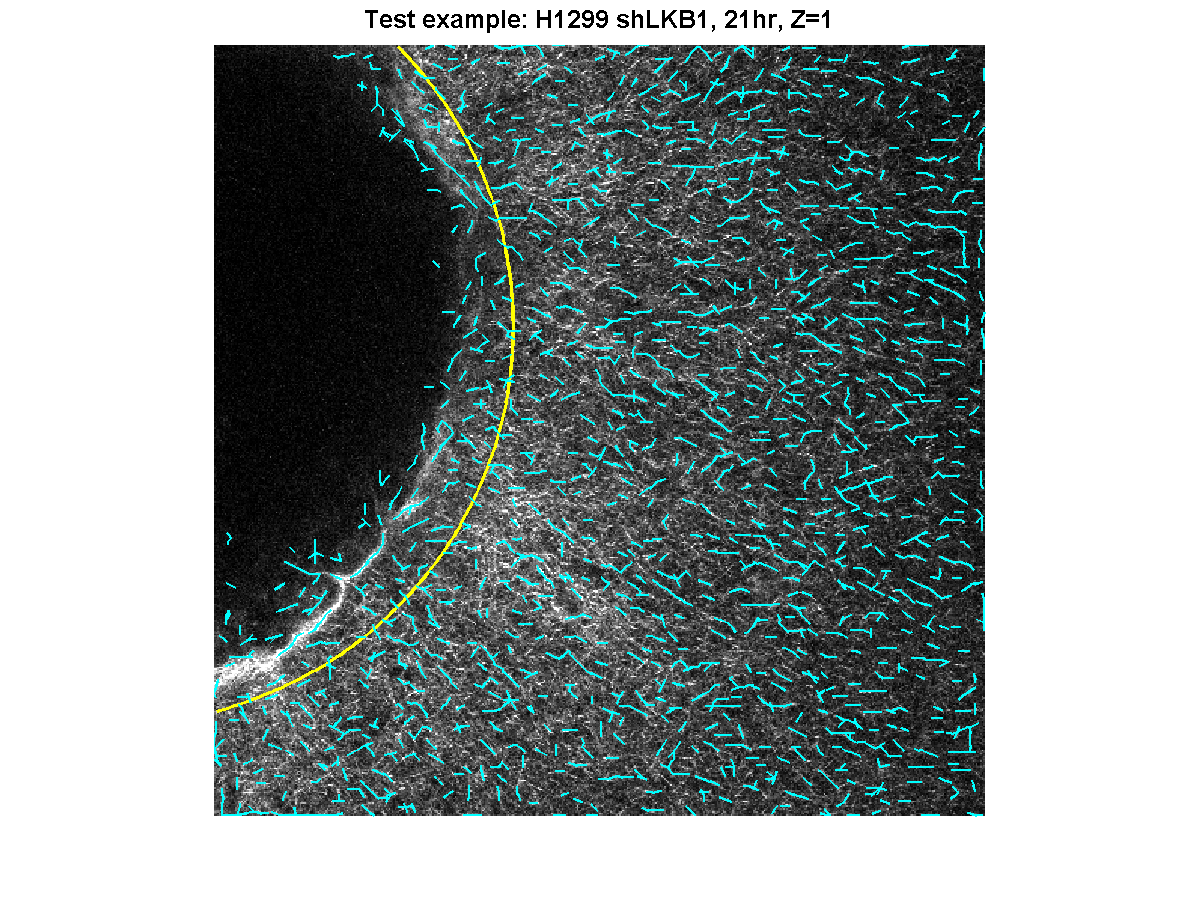

Supplement: Supplementary Dataset [file srep39498-s1.zip › Step1_Fig3b.tif]
