# Supplementary material for: Local alignment vectors reveal cancer cell-induced ECM fiber remodeling dynamics
Source: Sci Rep. 2017 Jan 3;7:39498. doi: 10.1038/srep39498 (PMC5206731; doi:10.1038/srep39498)
Supplement: Supplementary Information [file srep39498-s10.pdf]

Supplementary notes

Local alignment vectors reveal cancer cell-induced ECM fiber remodeling dynamics

Byoungkoo Lee[1], Jessica Konen[2,3], Scott Wilkinson[2,3], Adam I Marcus[3], and Yi Jiang[1]

[1] Department of Mathematics and Statistics, Georgia State University, Atlanta, GA, USA

[2] Cancer Biology Graduate Program, Emory University, Atlanta, GA, USA

[3] Department of Hematology and Oncology, Emory University, Atlanta, GA, USA

Finding optimal local circle size

Using NSCLC SHG images, where the image size is 512 x 512 pixel, we calculated alignment vectors for the seven different local circle sizes (radius of a local circle, R= 5, 10, 15, 20, 25, 30, 35 pixel). The smallest local circle (R=5) contains only a few quantized fiber segments, and thus its alignment is very high, closed to 1. As the local circle size increases, its alignment rapidly decreases from R=5 to R=25, because larger local circle contain more fiber segments to produce more reliable alignment vector. The alignment slowly decreases from R=25 to R=35, indicating that local alignment vectors lose their local heterogeneous fiber alignment feature and approach to the global fiber alignment vector. Therefore, we select R=25, the small enough size to address local fiber alignment pattern but large enough to avoid the sensitivity problem of small fiber segment count. Moreover, some z stack fiber images show only a few fibers in a large pixel area, so we add the minimum fiber constraint by excluding a local alignment vector, if its local circle contains less than or equal to 10 quantized fiber segments.

Supplementary Software: Local alignment vector software to demonstrate the local alignment vector method. Matlab codes and one example image data to produce Fig. 3 are included.

Supplementary Figure 1. Fiber alignment direction of *in silico* fibers using two different methods: alignment vector using Circular Statistics *vs*. average angle of the angular histogram. The simulation box is 512 x 512, and the length of each fiber is 40, with arbitrary unit. Initially all fibers are perfectly aligned to a specific direction (a) 90 degree (vertical), (b) 45 degree (diagonal), and (c) 0 degree (horizontal direction), and perturb the direction by adding an angle, sampled from normal distribution, where the mean is equal to zero and the standard deviation (std) is 30 degree. Total 100 fibers were sampled for each test case. The first row in (a,b,c) shows 100 *in silico* fibers, and the second row shows the fiber angular histogram, where the reference angle (0 degree) is the right horizontal direction and all fiber angles from 180 to 360 degree are subtracted by 180 degree. The third row shows fiber angular vectors mapping on the unit circle (open red circles), mean resultant vector direction (blue), and the alignment vector (green). The representative fiber alignment direction in angle (alignment vector vs average angle of the histogram) for each case is 98.2 vs 97.1 for (a), 42.5 vs 58.0 for (b), and 3.1 vs 74.6 for (c) in degree. The alignment vector angle properly represents

the fiber alignment direction, while the average angle of the angular distribution does not.

Supplementary Figure 2. Local alignment and quantized fiber segments count of H1299 pLKO.1 and shLKB1 at three different time points (0, 6, 21hr) after seeding the tumor spheroid into a 2mg/ml collagen gel for seven different local circle sizes (radius of local circle = 5, 10, 15, 20, 25, 30, 35 pixel), calculated using all z stack images.

Supplementary Figure 3. Local alignment and quantized fiber segments count of H157 $LKB1^{WT}$, Empty GFP (LKB1-null), $LKB1^{CTD}$ (C-terminal domain only) at two different time points (0, 24hr) after seeding the tumor spheroid into a 2mg/ml collagen gel for seven different local circle sizes (radius of local circle = 5, 10, 15, 20, 25, 30, 35 pixel), calculated using all z stack images.

Supplementary Figure 4. Local alignment and quantized fiber segments count of H157 $LKB1^{C430S}$(farnesylation mutant), $LKB1^{K78I}$ (kinase-dead mutant), and $LKB1^{K78I+C430S}$ (double mutant in kinase domain and farnesylation domain) cases at two different time points (0, 24hr) after seeding the tumor spheroid into a 2mg/ml collagen gel for seven different local circle sizes (radius of local circle = 5, 10, 15, 20, 25, 30, 35 pixel), calculated using all z stack images.

Supplementary Figure 5. Local alignment and quantized fiber segments count of H157 $LKB1^{WT}$+ $siRNA^{ctrl}$ and $LKB1^{WT}$+ $siRNA^{MARK1}$ cases at two different time points (0, 24hr) after seeding the tumor spheroid into a 2mg/ml collagen gel for seven different local circle sizes (radius of local circle = 5, 10, 15, 20, 25, 30, 35 pixel), calculated using all z stack images.

Supplementary Figure 6. Strongly aligned collagen volume ratio over all collagen volume for H1299 pLKO.1 and shLKB1 at three different time points (0, 6, 21hr). The percent of high alignment for five different thresholds (alignment > 0.5, …, 0.9) were calculated.

Supplementary Figure 7. Strongly aligned collagen volume ratio over all collagen volume for H157 $LKB1^{WT}$, Empty GFP (LKB1-null), $LKB1^{CTD}$ (C-terminal domain only), $LKB1^{C430S}$(farnesylation mutant), $LKB1^{K78I}$ (kinase-dead mutant), and $LKB1^{K78I+C430S}$ (double mutant in kinase domain and farnesylation domain), $LKB1^{WT}$+ $siRNA^{ctrl}$, and $LKB1^{WT}$+ $siRNA^{MARK1}$ at two different time points (0, 24hr). The percent of high alignment for five different thresholds (alignment > 0.5, …, 0.9) were calculated.

Video 1. The second harmonic generation microscopy images and quantized fiber segments of H1299 pLKO.1 and shLKB1 for three different time points (0, 6, 21hr). Eight z stack images were sampled from the total z stack to reduce the video size. Z = 1 for the bottom, and Z = 25 (pLKO.1, 0hr), 44 (pLKO.1, 6hr), 38 (pLKO.1, 21hr), 55 (shLKB1, 0hr), 45 (shLKB1, 6hr), 76 (shLKB1, 21hr) for the top of tumor spheroid, embedded in a 2mg/ml collagen gel. The z stack depth interval is 1μm, and the image is 512 x 512 pixel and 1pixel is 0.83 μm.

Video 2. Rotating view of local alignment vectors in 3D of H1299 pLKO.1 and shLKB1 for three different time points (0, 6, 21hr) using all z stack images. To more clearly visualize, only high alignment vectors (alignment >0.8, color-coded) are displayed with a fixed 25 pixel length, and z stack is scaled up by 4 times, e.g. X:Y:Z=1:1:4.

Video 3. The second harmonic generation microscopy images and quantized fiber segments of H157 $LKB1^{WT}$, Empty GFP (LKB1-null), $LKB1^{CTD}$ (C-terminal domain only) for two different time points (0, 24hr). Eight z stack images were sampled from the total z stack to reduce the video size. Z = 1 for the bottom, and Z = 8 ($LKB1^{WT}$, 0hr), 22 ($LKB1^{WT}$, 24hr), 24 (Empty GFP, 0hr), 93 (Empty GFP, 24hr), 10 ($LKB1^{CTD}$, 0hr), 55 ($LKB1^{CTD}$, 24hr) for the top of tumor spheroid, embedded in a 2mg/ml collagen gel. The z stack depth interval is 1μm, and the image is 512 x 512 pixel and 1pixel is 0.83 μm.

Video 4. Rotating view of local alignment vectors in 3D of H157 $LKB1^{WT}$, Empty GFP (LKB1-null), $LKB1^{CTD}$ (C-terminal domain only) for two different time points (0, 24hr) using all z stack images. To more clearly visualize, only high alignment vectors (alignment >0.8, color-coded) are displayed with a fixed 25 pixel length, and z stack is scaled up by 4 times, e.g. X:Y:Z=1:1:4.

Video 5. The second harmonic generation microscopy images and quantized fiber segments of H157 $LKB1^{C430S}$ (farnesylation mutant), $LKB1^{K78I}$ (kinase-dead mutant), and $LKB1^{K78I+C430S}$ (double mutant in kinase domain and farnesylation domain) cases at two different time points (0, 24hr). Eight z stack images were sampled from the total z stack to reduce the video size. Z = 1 for the bottom, and Z = 11 ($LKB1^{C430S}$, 0hr), 34 ($LKB1^{C430S}$, 24hr), 20 ($LKB1^{K78I}$, 0hr), 80 ($LKB1^{K78I}$, 24hr), 21 ($LKB1^{K78I+C430S}$, 0hr), 50 ($LKB1^{K78I+C430S}$, 24hr) for the top of tumor spheroid, embedded in a 2mg/ml collagen gel. The z stack depth interval is 1μm, and the image is 512 x 512 pixel and 1pixel is 0.83 μm.

Video 6. Rotating view of local alignment vectors in 3D of H157 $LKB1^{C430S}$ (farnesylation mutant), $LKB1^{K78I}$ (kinase-dead mutant), and $LKB1^{K78I+C430S}$ (double mutant in kinase domain and farnesylation domain) for two different time points (0, 24hr) using all z stack images. To more clearly visualize, only high alignment vectors (alignment >0.8, color-coded) are displayed with a fixed 25 pixel length, and z stack is scaled up by 4 times, e.g. X:Y:Z=1:1:4.

Video 7. The second harmonic generation microscopy images and quantized fiber segments of H157 $LKB1^{WT}$+ $siRNA^{ctrl}$ and $LKB1^{WT}$+ $siRNA^{MARK1}$ cases at two different time points (0, 24hr). Eight z stack images were sampled from the total z stack to reduce the video size. Z = 1 for the bottom, and Z = 20 ($LKB1^{WT}$+ $siRNA^{ctrl}$, 0hr), 26 ($LKB1^{WT}$+ $siRNA^{ctrl}$, 24hr), 15 ($LKB1^{WT}$+ $siRNA^{MARK1}$, 0hr), 52 ($LKB1^{WT}$+ $siRNA^{MARK1}$, 24hr) for the top of tumor spheroid, embedded in a 2mg/ml collagen gel. The z stack depth interval is 1μm, and the image is 512 x 512 pixel and 1pixel is 0.83 μm.

Video 8. Rotating view of local alignment vectors in 3D of H157 $LKB1^{WT}$+ $siRNA^{ctrl}$ and $LKB1^{WT}$+ $siRNA^{MARK1}$ for two different time points (0, 24hr) using all z stack images. To more clearly visualize, only high alignment vectors (alignment >0.8, color-coded) are displayed with a fixed 25 pixel length, and z stack is scaled up by 4 times, e.g. X:Y:Z=1:1:4.

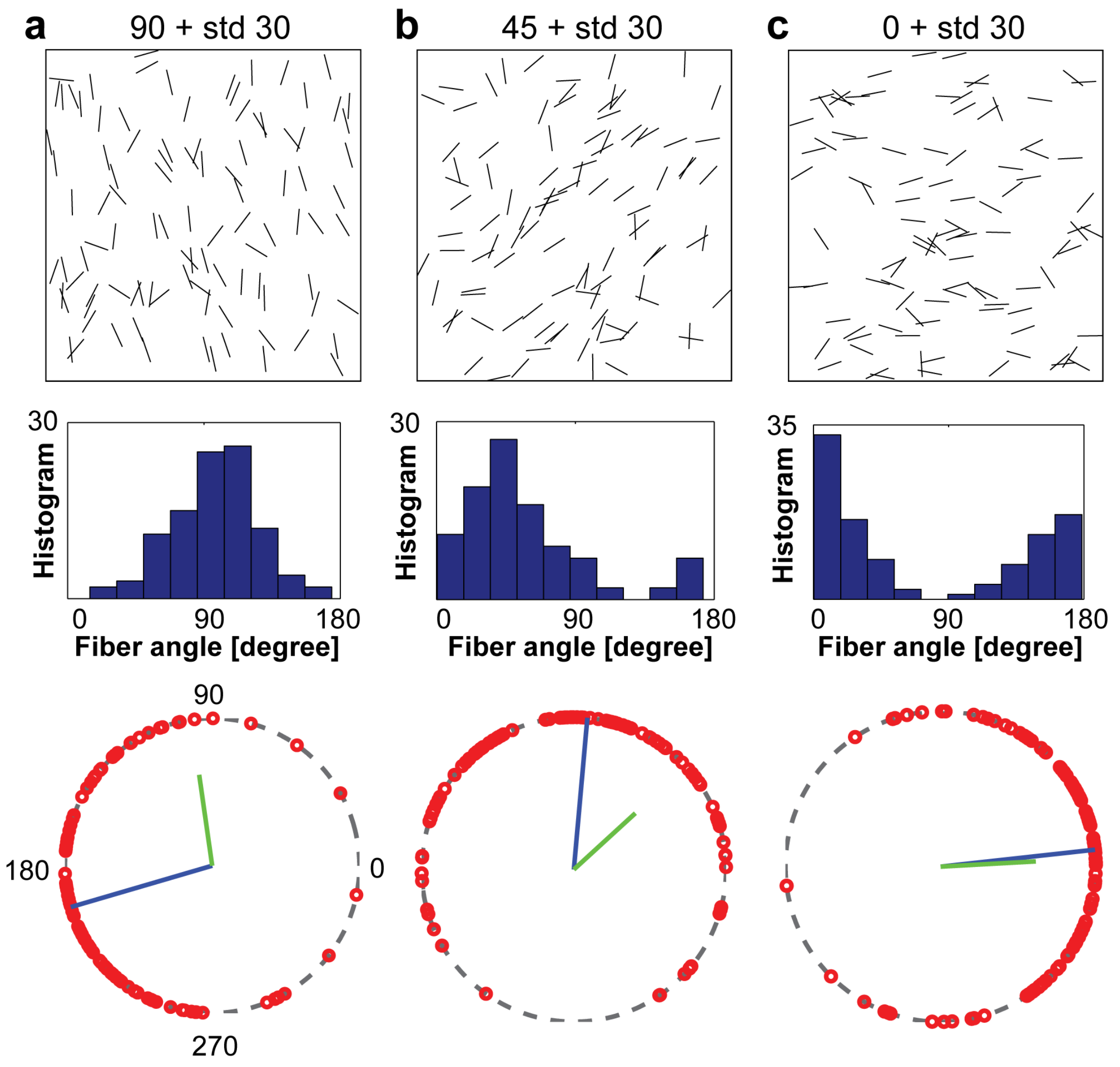

Lee et al., Supplementary Figure 1

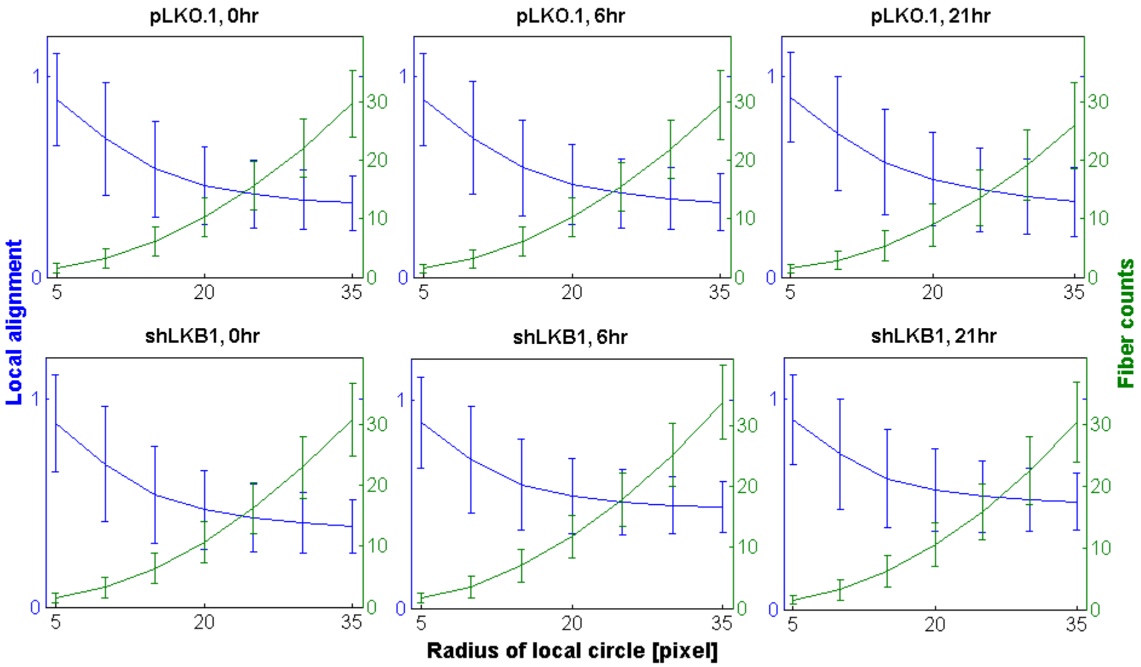

Lee et al., Supplementary Figure 2

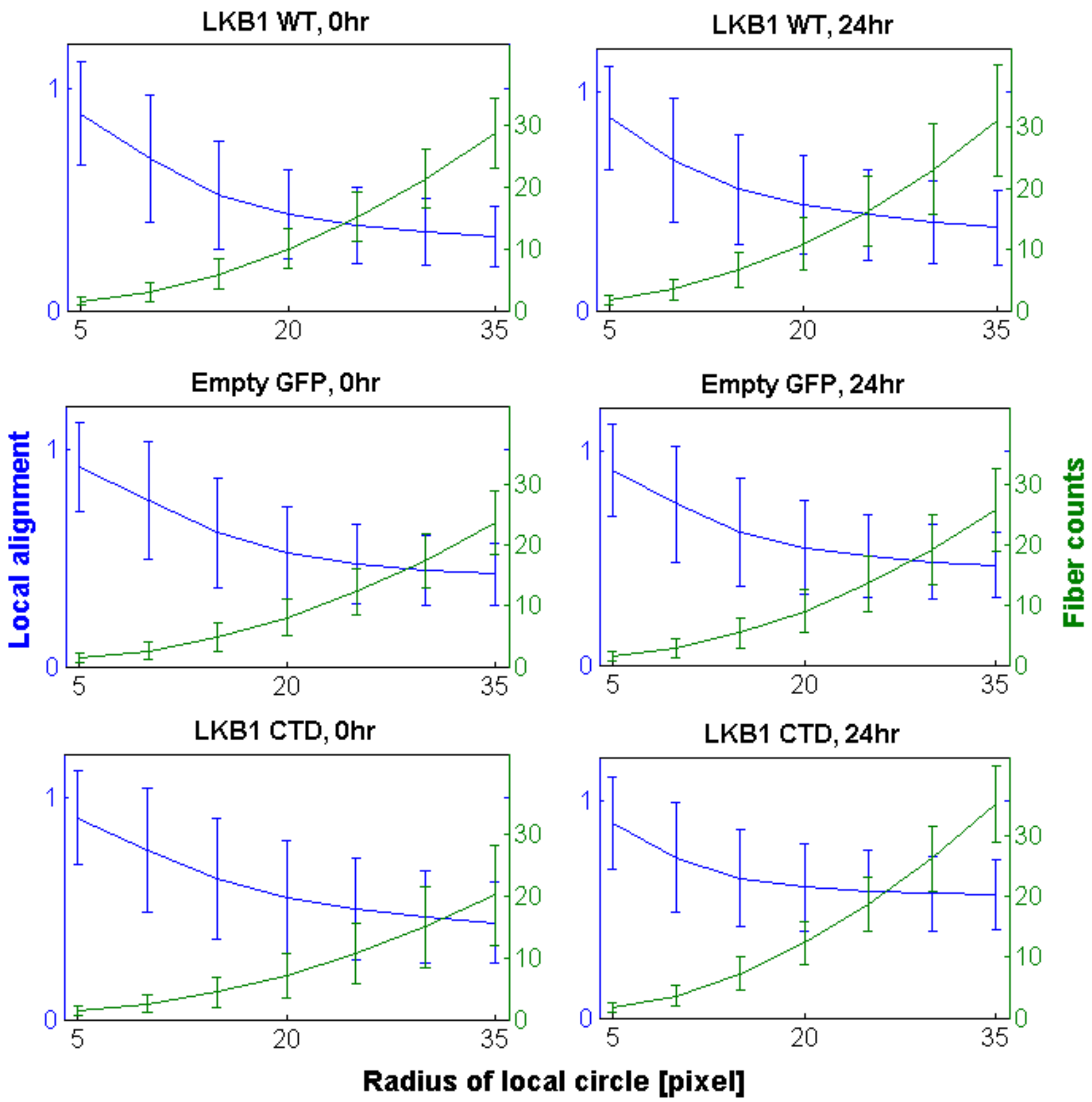

Lee et al., Supplementary Figure 3

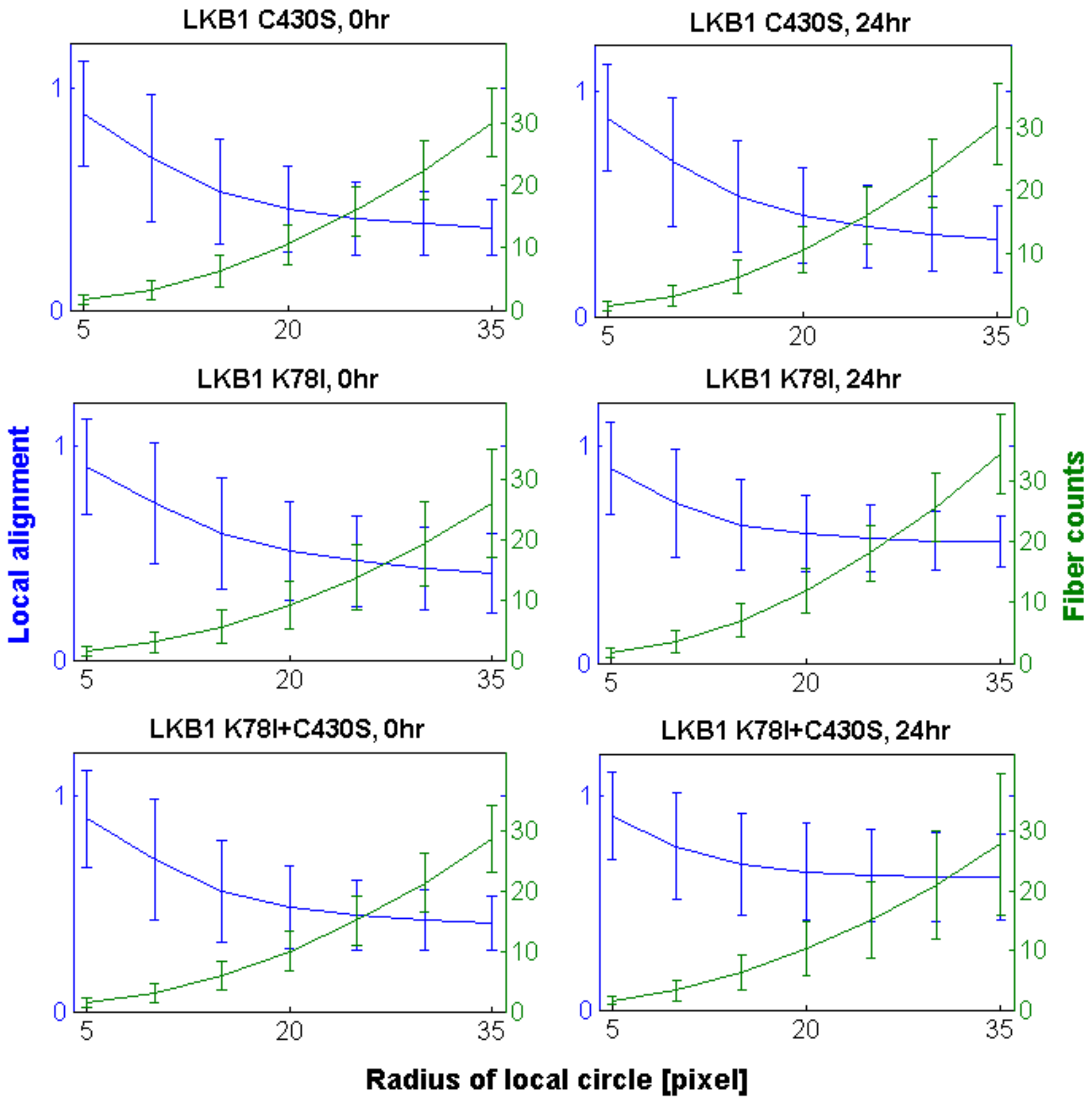

Lee et al., Supplementary Figure 4

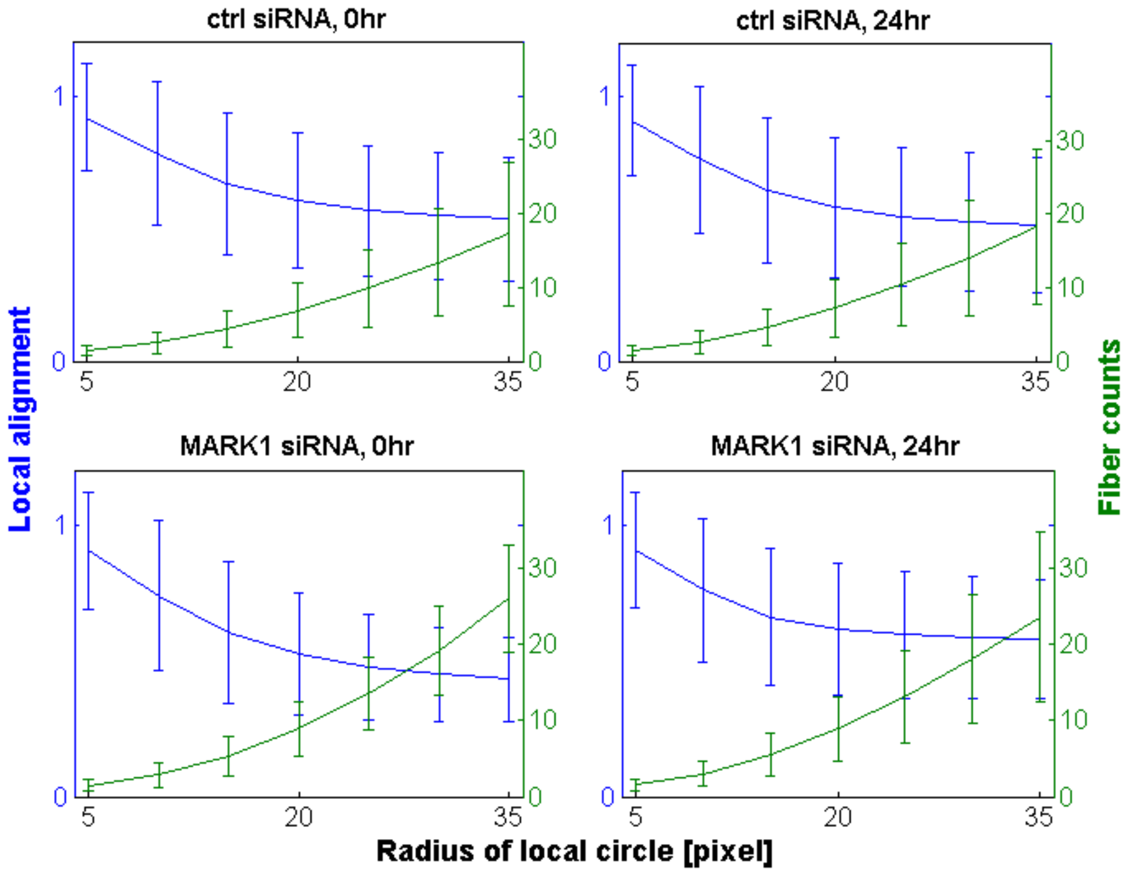

Lee et al., Supplementary Figure 5

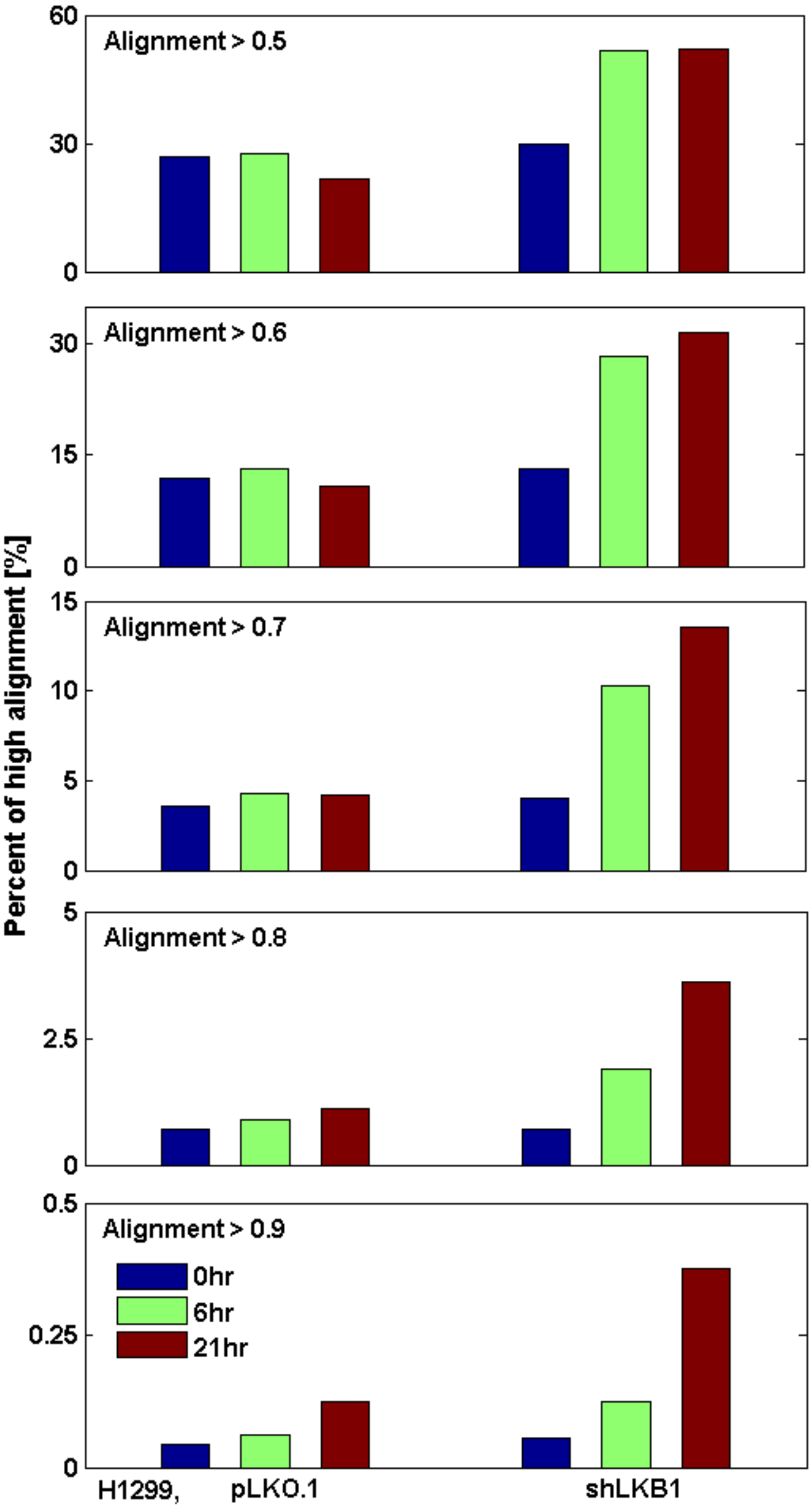

Lee et al., Supplementary Figure 6

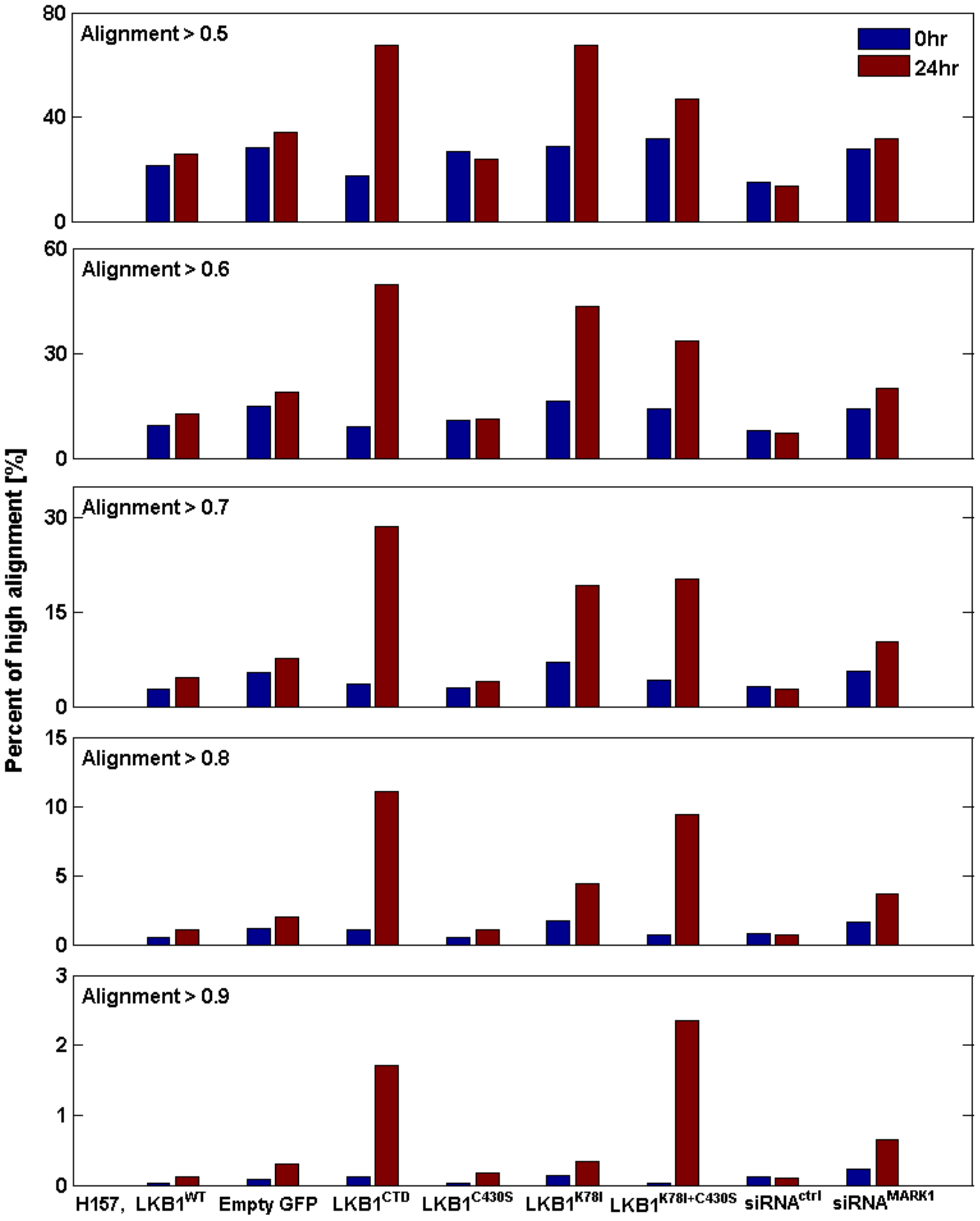

Lee et al., Supplementary Figure 7
